# Depletion of Amoxicillin and Its Major Metabolites in Anatolian Water Buffalo Milk After Intramuscular Administration

**DOI:** 10.3390/ani16060963

**Published:** 2026-03-19

**Authors:** Ulas Acaroz, Abdullah Eryavuz, Damla Arslan-Acaroz, Sinan Ince, Ibrahim Durmus, Azra Mila Eryavuz, Ismail Kucukkurt

**Affiliations:** 1 Department of Food Hygiene and Technology, Faculty of Veterinary Medicine, Afyon Kocatepe University, 03200 Afyonkarahisar, Türkiye; 2 Department of Food Hygiene and Technology, Faculty of Veterinary Medicine, Kyrgyz-Turkish Manas University, Bishkek 720038, Kyrgyzstan; 3 Department of Physiology, Faculty of Veterinary Medicine, Afyon Kocatepe University, 03200 Afyonkarahisar, Türkiye; eryavuz@aku.edu.tr; 4 Department of Biochemistry, Faculty of Veterinary Medicine, Afyon Kocatepe University, 03200 Afyonkarahisar, Türkiye; darslan@aku.edu.tr (D.A.-A.); kurt@aku.edu.tr (I.K.); 5 Department of Pharmacology and Toxicology, Faculty of Veterinary Medicine, Afyon Kocatepe University, 03200 Afyonkarahisar, Türkiye; since@aku.edu.tr; 6 Suhut Vocational School, Afyon Kocatepe University, 03800 Suhut, Türkiye; idurmus@aku.edu.tr; 7 Department of Biochemistry, Faculty of Veterinary Medicine Ceyhan, Çukurova University, 01950 Ceyhan Adana, Türkiye; aeryavuz@cu.edu.tr

**Keywords:** amoxicillin, water buffalo milk, drug residues, depletion kinetics, milk withdrawal period, metabolites, liquid chromatography–tandem mass spectrometry

## Abstract

Antibiotic residues in water buffalo milk are a food-safety concern, yet depletion data are scarce. The purpose of this study was to characterize the depletion profiles of amoxicillin (AMOX) and its two major metabolites, amoxicilloic acid (AMA) and amoxicillin diketopiperazine-2′,5′-dione (2,5-DKP), in Anatolian water buffalo milk after a single intramuscular administration and to estimate a milk withdrawal time relative to the EU MRL. We tested the hypothesis that AMOX concentrations would decrease below the EU MRL over successive milkings and that AMA and 2,5-DKP would exhibit depletion kinetics distinct from the parent compound. Five lactating Anatolian water buffaloes received a single intramuscular injection of amoxicillin (15 milligrams per kilogram). Milk was collected at each milking (twice daily) for seven days and analyzed by liquid chromatography–tandem mass spectrometry with quantification limits below the European Union maximum residue limit for amoxicillin in milk (4 micrograms per kilogram). Amoxicillin peaked at the second milking (mean 13.65 micrograms per kilogram), mean concentrations fell below the maximum residue limit from the sixth milking, and they became non-quantifiable from the tenth milking onward. Two major metabolites, amoxicillinic acid and amoxicillin diketopiperazine-2′,5′-dione, peaked earlier (2,5-DKP Tmax 12 h) or at higher concentrations (AMA Cmax 32.64 µg/kg vs. AMOX 13.65 µg/kg) and remained detectable up to the thirteenth milking, with longer apparent terminal half-lives (32.0 and 52.8 h) than amoxicillin (23.5 h); the mixed-effects model confirmed different depletion rates among analytes (milking × analyte interaction *p* = 4.63 × 10^−5^). A log-linear withdrawal model applying the EMA 95/95 tolerance limit indicated that the first time point at which the upper tolerance limit fell below the EU MRL was 84.7 h after dosing; rounded up to the next 12 h milking interval, this corresponds to a reported withdrawal period of 96 h (≈8 milkings). These results provide species-specific residue kinetics for amoxicillin in Anatolian buffalo milk and support considering metabolites in monitoring and withdrawal-time decisions.

## Simple Summary

Antibiotics can be necessary to treat infections in dairy animals, but traces of these drugs can pass into milk if it is collected too soon after treatment. Water buffalo are an important milk species worldwide, yet there is limited information on how quickly some antibiotics leave buffalo milk. In this study, five lactating Anatolian water buffaloes received one intramuscular injection of amoxicillin, and milk was sampled at every milking for seven days. We measured amoxicillin and two major breakdown products using a highly sensitive laboratory method. Amoxicillin was highest at the second milking and then decreased; mean concentrations fell below the European Union MRL by around the sixth milking and were below the LOD after the tenth milking. Because one individual animal was slightly above the MRL at the sixth milking (4.2 µg/kg), all individual samples were below the MRL from the seventh milking onward. The two breakdown products persisted longer than AMOX, remaining detectable up to about the thirteenth milking, whereas AMOX was no longer detectable from the tenth milking onward. Based on the EMA-recommended 95/95 tolerance limit approach, the withdrawal time corresponded to 84.7 h after dosing; when rounded up to the next 12 h milking interval, this gives a practical withdrawal period of 96 h (≈8 milkings). This evidence can help farmers, veterinarians, and regulators set practical waiting times for buffalo milk and suggests that breakdown products may also be useful to track in safety testing.

## 1. Introduction

Milk is one of the most widely consumed animal-derived foods and contributes substantially to daily energy and nutrient intake worldwide. It provides high-quality proteins, bioavailable calcium and phosphorus, fat-soluble vitamins and a range of bioactive lipids, and its regular consumption has been associated with beneficial effects on bone health, cardiometabolic outcomes and overall diet quality [1,2,3,4,5]. Because milk and dairy products are consumed frequently across all age groups—including children and other vulnerable consumers—chemical hazards in milk, even at low concentrations, are of particular public health relevance.

Antimicrobial agents remain indispensable for maintaining animal welfare and productivity in dairy herds, especially for the treatment of mastitis. However, their use may lead to drug residues entering the milk chain when withdrawal periods are not fully respected, or when depletion kinetics are altered by disease status, dosing regimen, formulation, and milking management [6,7]. From a human health perspective, exposure to antibiotic residues has been associated with hypersensitivity reactions (particularly for β-lactams), potential disruption of the intestinal microbiota, and—most importantly in a One Health context—the selection and dissemination of antimicrobial resistance (AMR) [6,8]. Beyond direct toxicological concerns, residues can also compromise dairy fermentations by inhibiting starter cultures, thereby affecting product quality and causing economic losses; importantly, this may occur even when residue levels are within legally admissible limits [7,9]. In addition, antibiotic residues have been detected in raw milk samples collected during and even after the labelled withdrawal period in real farming conditions, underlining the need for robust residue monitoring and species-appropriate withdrawal times [10].

Among the different dairy species, the water buffalo (*Bubalus bubalis*) plays an important role in global milk production, with the vast majority of buffalo milk produced in warm-climate regions—predominantly in Asia—and with production also extending to other regions including parts of the Mediterranean basin and South America; buffalo milk is consumed as drinking milk and is widely used as a raw material for high-value cheeses and traditional dairy products [11,12,13]. According to FAO statistics, cattle produce about 81–83% of global milk production, followed by buffaloes with roughly 13–15%, goats with around 2% and sheep with around 1%, while camels contribute less than 1% [14,15]. Buffalo milk is clearly the second most produced milk type worldwide; recent global analyses indicate that buffalo milk accounts for around 15% of total milk output, confirming its strategic importance in the dairy sector [16,17]. Buffalo milk is chemically characterized by a higher concentration of major constituents than cow milk, typically containing approximately 6–8% fat and approximately 4–5% protein, with lactose generally around 4.7–4.9% and total solids around 15–17% [11,12]. For example, a large dataset of buffalo milk samples reported seasonal mean values of fat of approximately 6.01–6.31 g/100 g, protein approximately 4.18–4.23 g/100 g, lactose approximately 4.75–4.76 g/100 g, and total solids approximately 15.95–16.23 g/100 g, illustrating the dense nutrient matrix of buffalo milk [11]. Such compositional differences may influence drug distribution between aqueous and fat/protein phases and thereby affect residue depletion in milk. Despite the strategic importance of buffalo milk for human nutrition and dairy processing, buffalo production systems remain under-represented in many residue datasets.

β-lactam antibiotics are among the most frequently used drug classes in dairy practice because of their broad spectrum of activity and favourable safety profile. Amoxicillin (AMOX), a semi-synthetic aminopenicillin, is widely administered in both veterinary and human medicine for the treatment of mastitis and systemic infections in bovines and other food-producing species [18,19]. Its bactericidal effect is mediated by inhibition of cell wall synthesis through binding to penicillin-binding proteins and activation of autolytic enzymes [20]. In the European Union, the MRL for AMOX in milk is set at 4 µg/kg under Commission Regulation (EU) No 37/2010, and label withdrawal periods are established to ensure that residues fall below this limit [21]. Nevertheless, β-lactam residues continue to be reported in raw milk in different regions, suggesting that deviations in dosing regimens, formulations or milking intervals, as well as species- and system-specific factors, may influence depletion kinetics [22,23,24].

After administration, AMOX undergoes biotransformation/degradation to several products, which include amoxicillinic (amoxicillolic) acid (AMA) and amoxicillin diketopiperazine-2′,5′-dione (2,5-DKP). These products arise primarily through β-lactam ring opening followed by rearrangement and/or intramolecular cyclization, and their formation can be influenced by matrix conditions such as pH [25,26,27]. Because transformation products may show different stability and depletion behaviors than the parent drug, monitoring AMA and 2,5-DKP alongside AMOX can provide a more complete picture of residue kinetics and can strengthen withdrawal-period assessment. Moreover, AMOX transformation products have been discussed in the context of hyper-sensitivity mechanisms (e.g., hapten formation), highlighting the relevance of metabolite monitoring in consumer-risk assessment [27].

Liquid chromatography coupled with tandem mass spectrometry (LC–MS/MS) has become the method of choice for confirmatory analyses of veterinary drug residues in milk because it provides high sensitivity and selectivity in complex matrices. Recent multi-residue workflows have reported low-µg/kg quantification of β-lactams in milk and other food matrices [24,28]. In parallel, targeted LC–MS/MS approaches continue to be developed for the simultaneous determination of AMOX and major transformation products (e.g., AMA and 2,5-DKP) in biological matrices [29]. However, there is still a paucity of data describing the time-course of AMOX, AMA and 2,5-DKP in the milk of water buffaloes (*Bubalus bubalis*). To date, most buffalo-focused depletion studies have addressed other antimicrobials such as kanamycin, enrofloxacin and tylosin in Anatolian buffalo milk [30,31,32], leaving a clear gap regarding β-lactam excretion kinetics in this species.

Anatolian water buffaloes represent an important local genetic resource and contribute to regional dairy production; yet, to our knowledge, there are no published data describing the milk excretion profile of AMOX together with its major metabolites (AMA and 2,5-DKP) in this species. This lack of species-specific information is not merely academic: from a consumer-protection perspective, withdrawal periods are only as reliable as the depletion data that support them, and depletion behavior may differ between dairy species because of differences in milk composition, udder physiology and drug disposition.

Accordingly, this study aimed to quantify AMOX, AMA, and 2,5-DKP in Anatolian water buffalo milk using a validated LC–MS/MS method and to characterize their depletion profiles following a single intramuscular dose. We hypothesized that depletion kinetics in buffalo milk would differ from bovine patterns due to the richer milk matrix, and that AMA and 2,5-DKP could persist longer than the parent compound, so that monitoring AMOX alone may underestimate AMOX-related residues at later time points. These data are intended to support evidence-based, species-appropriate withdrawal recommendations for dairy buffalo milk.

## 2. Materials and Methods

### 2.1. Ethical Statement

Ethical approval (Approval No: 49533702/97) was given to this study by Afyon Kocatepe University Animal Experiments Local Ethics Committee.

### 2.2. Experimental Animals

This study was conducted during the summer season (August) in five clinically healthy Anatolian buffaloes (400–500 kg body weight) at the Research and Application Farm of Afyon Kocatepe University, Afyonkarahisar, Türkiye. The animals were maintained in a semi-open barn management system under routine farm conditions. Detailed information regarding age, parity, lactation stage of the animals is presented in Table S1. Each animal had unrestricted access to drinking water and received a standard lactation ration as detailed in Table S2. Prior to the drug administration, blank milk samples were collected. To reflect routine on-farm treatment conditions, amoxicillin was administered as a commercially available long-acting injectable veterinary medicinal product containing amoxicillin trihydrate as the active ingredient and formulation excipients/coformulants (VilamoksLA, Vilsan, Ankara, Türkiye) at a single intramuscular dose of 15 mg/kg. After AMOX administration, milk samples were collected at 12 h intervals (i.e., twice daily) for 7 days. Following collection, milk samples were preserved at −20 °C until further analytical procedures.

### 2.3. Chemicals

Amoxicillin (AMOX; 99.4% purity, specified on an anhydrous basis) was obtained from Sigma-Aldrich (St. Louis, MO, USA). Its metabolites, amoxicilloic acid (AMA, 95%) and amoxicillin diketopiperazine (2,5-DKP, >95%), were purchased from Santa Cruz Biotechnology, Inc. (Dallas, TX, USA). All remaining chemicals and reagents used in the study were of analytical grade and obtained from commercial suppliers.

### 2.4. Sample Extraction

The extraction of AMOX and its primary metabolites, AMA and 2,5-DKP, from buffalo milk samples was carried out with a modified version of the protocol described by Jank et al. [28]. In brief, two milliliters of each milk sample were placed into polystyrene centrifuge tubes and mixed with 4 mL of acetonitrile using a vortex mixer. The mixture was subjected to triple centrifugation at 4000 rpm for 10 min at 4 °C, with final centrifugation at 2 °C to ensure maximum clarity. The supernatant was carefully collected and evaporated under a stream of nitrogen (N_2_) in a water bath maintained at ≤45 °C until the volume was reduced to approximately 500 µL. The resulting volume was adjusted to 1 mL using the mobile phase and transferred to HPLC vials for analysis.

### 2.5. LC Separation

Analysis of AMOX, AMA and 2,5-DKP was performed using an Agilent Technologies 1200 Series LC system (Agilent Technologies, Santa Clara, CA, USA). This system was coupled with an Agilent 6460 triple quadrupole mass spectrometer, which was equipped with an electrospray ionization (ESI) source (Waldbronn, Germany). Chromatographic separation was carried out on a ZORBAX RRHT Eclipse Plus C18 column (150 × 4.6 mm, 1.8 µm; Agilent Technologies, Santa Clara, CA, USA) maintained at 45 °C. The mobile phase consisted of solvent A (0.1% formic acid in ultrapure water) and solvent B (acetonitrile). The gradient elution program was as follows: 0.00–1.00 min, 100% A; 1.00–3.00 min, linear gradient to 20% A and 80% B; 3.00–7.50 min, linear gradient to 5% A and 95% B; 7.50–8.10 min, return to 100% A; 8.10–12.00 min, re-equilibration at 100% A. The flow rate was 0.6 mL/min and the injection volume was set to 20 µL.

### 2.6. Mass Spectrometry Parameters and Method Validation

The ESI source was operated in positive ion mode. Nebulization and drying gases were supplied by a nitrogen generator (Balston, Haverhill, MA, USA) at 350 °C. The MS parameters were as follows: sheath gas flow rate, 10 L/min; nebulizer pressure, 40 psi; capillary voltage, 4000 V; and sheath gas temperature, 400 °C. Precursor/product ion transitions were monitored in Multiple Reaction Monitoring (MRM) mode.

Retention times were 2.685 min for AMOX, 4.533 min for AMA and 5.156 min for 2,5-DKP. For AMOX, the precursor ion was determined as *m*/*z* 365 and the product ions were *m*/*z* 333 and 98. For AMA, the precursor ion was *m*/*z* 384.1 and the product ions were *m*/*z* 155.9 and 107.9. For the 2,5-DKP, the precursor ion is *m*/*z* 365.9 and the product ions are *m*/*z* 207 and 160.

Method validation was performed in raw buffalo milk according to VICH GL49 [33] to confirm the suitability of the method for residue depletion studies. Selectivity was evaluated by analyzing blank (pre-dose) buffalo milk samples to verify the absence of interfering peaks at the retention times and MRM transitions of AMOX, AMA and 2,5-DKP.

Matrix-matched calibration curves were prepared by fortifying blank buffalo milk at seven concentration levels (0.5, 1.0, 2.0, 5.0, 10.0, 20.0 and 50.0 µg/kg) and processing these samples through the complete extraction and LC–MS/MS procedure. Linearity was assessed by least-squares linear regression and expressed as the coefficient of determination (R^2^).

Limits of detection (LOD) and quantification (LOQ) were determined in matrix-matched spiked samples using signal-to-noise (S/N) criteria, defining LOD as the lowest level producing S/N ≥ 3 and LOQ as the lowest level producing S/N ≥ 10; LOQ was additionally required to meet acceptable accuracy and precision.

Accuracy (recovery) and precision were evaluated by spiking blank milk at three concentration levels (10, 20 and 50 µg/kg). Recovery was calculated as (measured concentration/fortified concentration) × 100 and reported as mean ± SD (%). Repeatability (intra-day) and within-laboratory reproducibility (inter-day) were expressed as relative standard deviation (RSD% = SD/mean × 100). Intra-day precision was evaluated within a single analytical run, whereas inter-day precision was assessed across independent analytical runs performed on different days. Quantification was performed on a parent-compound-equivalent basis. Concentration calculations were adjusted according to the purity specified on an anhydrous basis for the AMOX reference standard.

### 2.7. Statistical Analysis

LC–MS/MS raw data were processed using Agilent MassHunter Workstation (v7.0). Statistical analyses were performed in R (v4.5.1). Concentrations of AMOX, AMA and 2,5-DKP at each milking were summarized as mean ± SD (and min–max and coefficient of variation where appropriate).

To account for repeated measurements within the same animals, depletion profiles were analyzed using a linear mixed-effects model on log_10_-transformed concentrations (log_10_[Conc + 0.01]). Milking number, analyte (AMOX, AMA, 2,5-DKP) and their interaction were included as fixed effects, and animal identity was included as a random intercept. Type III tests with Satterthwaite’s approximation (lmerTest) were used for inference. A *p*-value < 0.05 was considered statistically significant.

Pearson correlation coefficients were calculated for AMOX–AMA and AMOX–2,5-DKP pairs using paired observations and reported with two-sided *p*-values. Non-compartmental parameters were derived for each animal and analyte: Cmax and Tmax were obtained directly from the observed data, and AUC_0_–t was calculated using the linear trapezoidal rule. Terminal half-life (t½) was estimated from the slope of the log-linear depletion phase (t½ = ln(2)/|slope|). The log-linear phase used for slope estimation was defined as the period covering all post-treatment milk samples with detectable concentrations (≥LOD) up to the last detected time point; pre-treatment blanks and non-detects (<LOD) were excluded because ln-transformation is not defined for zero values. For AMOX, this corresponded to milkings 1–9 (12–108 h) in the present dataset. The appropriateness of the log-linear assumption was verified by inspecting semi-log plots and regression diagnostics (linearity and residual patterns). Because samples were collected at 12 h intervals and concentrations followed a single log-linear decline over the detectable period, the same interval was used for both half-life estimation and withdrawal-time modeling.

For AMOX, milk withdrawal time was estimated from a log-linear regression of ln-transformed concentrations versus time after dosing (h). In line with EMA guidance for milk withdrawal periods, the withdrawal time was defined as the earliest time point at which the upper one-sided 95/95 tolerance limit (i.e., the upper 95% confidence limit of the 95th percentile of the population) fell below the EU MRL for AMOX in milk (4 µg/kg), in accordance with current EMA guidance [34]. For practical application under a 12 h milking scheme, the calculated withdrawal time was rounded up to the next full 12 h milking interval and reported in hours and as the corresponding number of milkings.

## 3. Results

### 3.1. Method Validation

**Figure 1:**
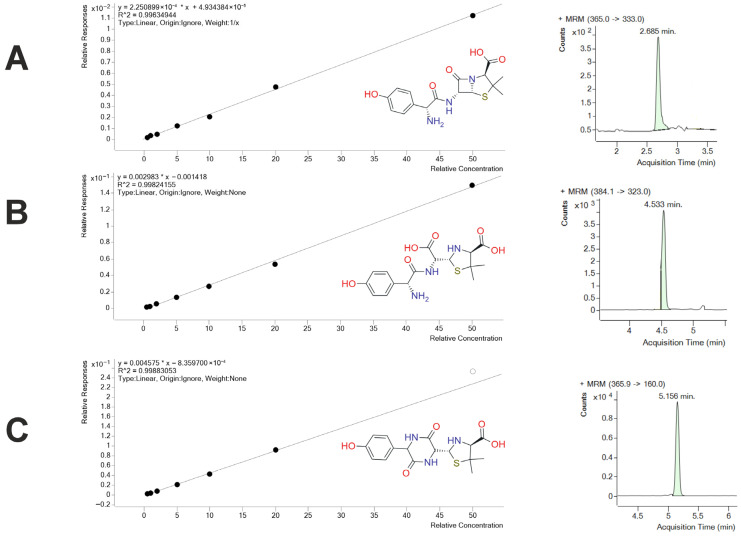
Calibration curves (left) and representative LC–MS/MS chromatograms (right) of target analytes in buffalo milk. (**A**) Amoxicillin (AMOX): calibration curve demonstrating excellent linearity (R^2^ > 0.99) and corresponding chromatogram showing a well-resolved peak at 2.685 min. (**B**) Amoxicillinic acid (AMA): calibration curve with high linearity (R^2^ > 0.99) and representative chromatogram with a retention time of 4.533 min. (**C**) Amoxicillin diketopiperazine-2′,5′-dione (2,5-DKP): calibration curve (R^2^ > 0.99) and chromatogram indicating a distinct peak at 5.156 min. Chemical structures were generated using Marvin 25.5.3 (ChemAxon, Budapest, Hungary) (https://chemaxon.com/marvin, accessed on 25 December 2025) based on molecular information retrieved from the PubChem database (National Center for Biotechnology Information, Bethesda, MD, USA). The black dots represent calibration data points used for constructing the calibration curves, while the green shaded areas indicate the integrated chromatographic peaks.

Quantitative determination of amoxicillin (AMOX) and its two major metabolites, amoxicillinic acid (AMA) and amoxicillin diketopiperazine-2′,5′-dione (2,5-DKP), in buffalo milk was achieved using an LC–MS/MS method. Matrix-matched calibration curves were prepared at seven concentration levels (0.5, 1.0, 2.0, 5.0, 10.0, 20.0 and 50.0 µg/kg). All three analytes showed excellent linearity across this range, with determination coefficients (R^2^) of 0.9963 for AMOX, 0.9982 for AMA and 0.9988 for 2,5-DKP. Representative LC–MS/MS chromatograms and calibration curves are presented in Figure 1. The chromatographic peaks were well resolved and symmetrical, confirming the reliability of the quantitative method.

Limits of detection (LOD) were 0.29 µg/kg for AMOX, 0.72 µg/kg for AMA and 0.85 µg/kg for 2,5-DKP, whereas the corresponding limits of quantification (LOQ) were 0.97, 2.41 and 2.83 µg/kg, respectively. These values are well below the maximum residue limit (MRL) of 4 µg/kg established for AMOX in milk by the European Union [21], indicating that the method is sufficiently sensitive for monitoring low residue levels in this high fat matrix.

Accuracy and precision were assessed by fortifying blank buffalo milk at 10, 20 and 50 µg/kg (*n* = 9 per level). Intra-day recovery values ranged from 90.41% to 96.72%, and inter-day recoveries from 86.26% to 94.88%. Relative standard deviations (RSDs) were below 7% for all analytes and concentration levels in both intra- and inter-day experiments (Table 1). Taken together, the high recoveries, low RSDs and low LOD/LOQ values demonstrate that AMOX, AMA and 2,5-DKP can be quantified in buffalo milk with high accuracy, precision and sensitivity. Comparative tables with other studies [35,36,37,38] are summarized in Supplementary Table S3.

### 3.2. Milk Depletion of Amoxicillin and Metabolites

**Figure 2:**
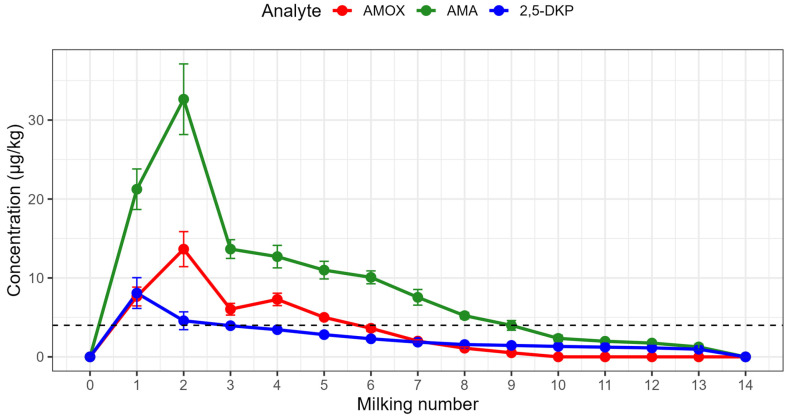
Mean concentration–time profiles of AMOX, AMA and 2,5-DKP in buffalo milk following intramuscular administration of AMOX (15 mg/kg) to five buffaloes. Data points represent mean concentrations at each milking (*n* = 5).

After intramuscular administration of AMOX to five Anatolian buffaloes, a total of 75 milk samples (5 animals × 15 milking times) were collected from the pre-treatment milking (0) to the 14th milking. None of the analytes were detected in pre-dose samples. Mean concentration–time profiles for AMOX, AMA and 2,5-DKP are shown in Figure 2, and individual AMOX profiles in Supplementary Figure S1; descriptive statistics for each time point are provided in Supplementary Table S4.

AMOX concentrations rose rapidly after dosing, reaching a mean (±SD) peak of 13.65 ± 2.22 µg/kg at the second milking (24 h). Thereafter, AMOX declined approximately mono exponentially. Mean concentrations decreased to 5.01 ± 0.20 µg/kg at the fifth milking and 3.62 ± 0.40 µg/kg at the sixth milking, the latter already below the EU MRL of 4 µg/kg. By the ninth milking, the mean AMOX level had fallen to 0.52 ± 0.07 µg/kg, and AMOX was not detectable in any animal from the tenth milking onwards (Supplementary Table S4; Figure 2 and Figure S1). In comparison, metabolite depletion was slower, supported by longer terminal half-lives (AMOX 23.5 h; AMA 32.0 h; 2,5-DKP 52.8 h; Table 2) and a significant milking × analyte interaction in the mixed-effects model (F_2,219_ = 10.45; *p* = 4.63 × 10^−5^; Table 3). AMA concentrations peaked at the same time as AMOX (second milking; Tmax 24 h) but at higher concentrations (Cmax 32.64 ± 4.47 vs. 13.65 ± 2.22 µg/kg; Table 2), whereas 2,5-DKP reached its maximum at the first milking (12 h; Tmax 12 h; Table 2). Both AMA and 2,5-DKP remained measurable (≥LOD) in milk up to the thirteenth milking, whereas AMOX was not detectable from the tenth milking onward (Supplementary Table S4). Consistent with its longer half-life, 2,5-DKP declined most gradually and persisted at low but detectable levels even when AMOX was no longer quantifiable.

Non-compartmental pharmacokinetic parameters derived from the concentration–time data are summarized in Table 2, and the corresponding individual Cmax, Tmax, and AUC_0_–t values for each buffalo are presented in Supplementary Table S5. Mean Cmax values were highest for AMA (32.64 ± 4.47 µg/kg), followed by AMOX (13.65 ± 2.22 µg/kg) and 2,5-DKP (8.09 ± 1.95 µg/kg) (Table 2). Across animals, AMOX Cmax ranged from 10.5 to 16.5 µg/kg, whereas AMA Cmax ranged from 28.16 to 39.77 µg/kg and 2,5-DKP Cmax from 6.01 to 10.80 µg/kg. The corresponding AUC_0_–t values showed the same pattern, with the highest overall exposure for AMA (1504.68 ± 139.68) and the lowest for 2,5-DKP (416.38 ± 44.24), while AMOX was intermediate (562.32 ± 58.65) (Table 2). Tmax was consistently 24 h for AMOX and AMA and 12 h for 2,5-DKP in all animals. The terminal elimination rate constants translated into mean half-lives of 23.5 h for AMOX, 32.0 h for AMA and 52.8 h for 2,5-DKP, confirming that the metabolites—especially 2,5-DKP—are cleared from milk more slowly than the parent drug.

### 3.3. Mixed-Effects Modeling

To formally evaluate the effects of time and analyte on milk concentrations, a linear mixed-effects model was fitted to log_10_ transformed concentrations, with milking number, analyte and their interaction as fixed effects and animal as a random intercept. The ANOVA of the fixed effects is presented in Table 3. Milking number had a highly significant effect on concentration (F_1,219_ = 52.36; *p* = 7.7 × 10^−12^), reflecting the overall decline of residues over time. The main effect of the analyte was of borderline significance (F_2,219_ = 2.68; *p* = 0.071), whereas the milking × analyte interaction was clearly significant (F_2,219_ = 10.45; *p* = 4.63 × 10^−5^). Thus, depletion rates differed among AMOX, AMA and 2,5-DKP, consistent with the longer terminal half-lives estimated for AMA and 2,5-DKP compared with AMOX (Table 2).

### 3.4. Relationship Between AMOX and Its Metabolites

**Figure 3:**
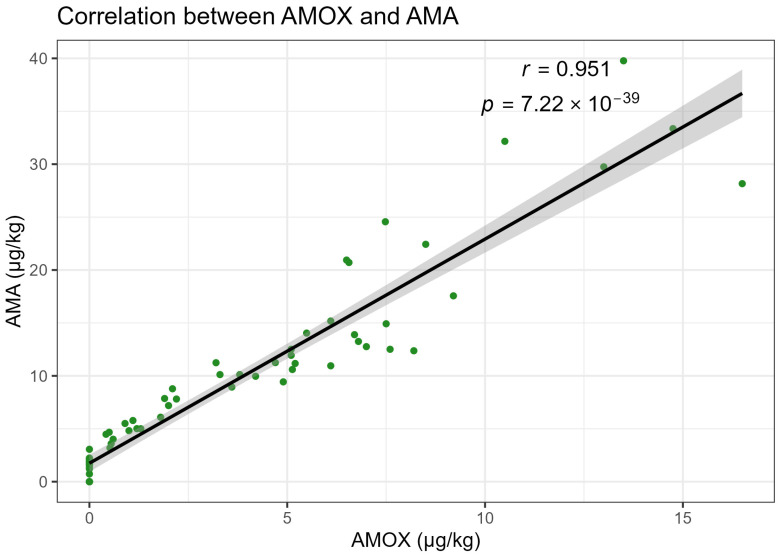
Scatter plot showing the strong positive correlation between AMOX and AMA concentrations in buffalo milk, with the fitted regression line and 95% confidence interval. The dots represent individual observations, and the shaded area indicates the 95% confidence interval of the fitted regression line. The Pearson correlation coefficient was r = 0.951 (*p* = 7.22 × 10^−39^).

**Figure 4:**
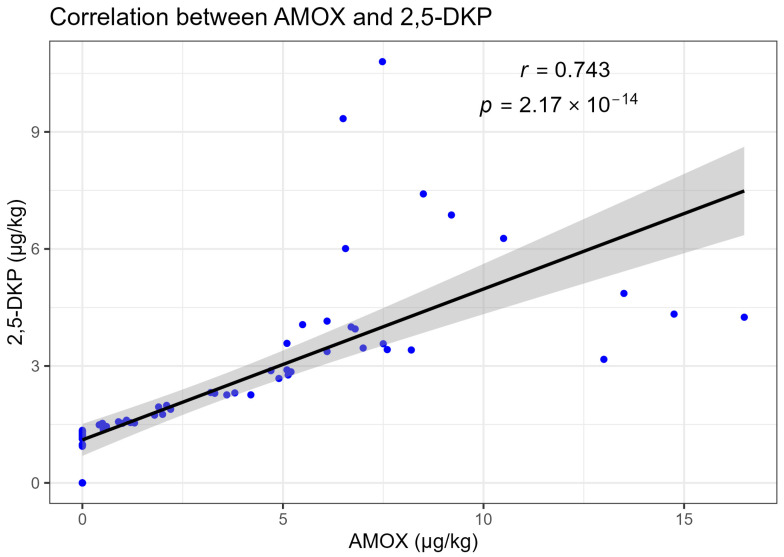
Scatter plot illustrating the positive correlation between AMOX and 2,5-DKP concentrations in buffalo milk, with the fitted regression line and 95% confidence interval. The dots represent individual observations, and the shaded area indicates the 95% confidence interval of the fitted regression line.The Pearson correlation coefficient was r = 0.743 (*p* = 2.17 × 10^−14^).

The concentrations of AMOX were strongly associated with those of its metabolites in milk. When data from all animals and sampling times were pooled, AMOX and AMA concentrations exhibited an almost linear relationship, with a Pearson correlation coefficient of r = 0.951 (*p* = 7.22 × 10^−39^; Figure 3). AMOX and 2,5-DKP showed a statistically significant positive correlation (r = 0.743, *p* = 2.17 × 10^−14^; Figure 4). The narrow confidence bands around the regression lines indicate limited scatter and suggest that, within the studied range, variations in metabolite concentrations closely mirror those of the parent drug.

### 3.5. Withdrawal-Time Estimation

Withdrawal time for AMOX in buffalo milk was estimated by fitting a log-linear regression to ln-transformed AMOX concentrations versus time after dosing (h). The resulting model, ln(C) = 3.03436 − 0.02945·t, described the data well (R^2^ = 0.85, *p* < 0.001), corresponding to an apparent terminal half-life of approximately 23.5 h.

**Figure 5:**
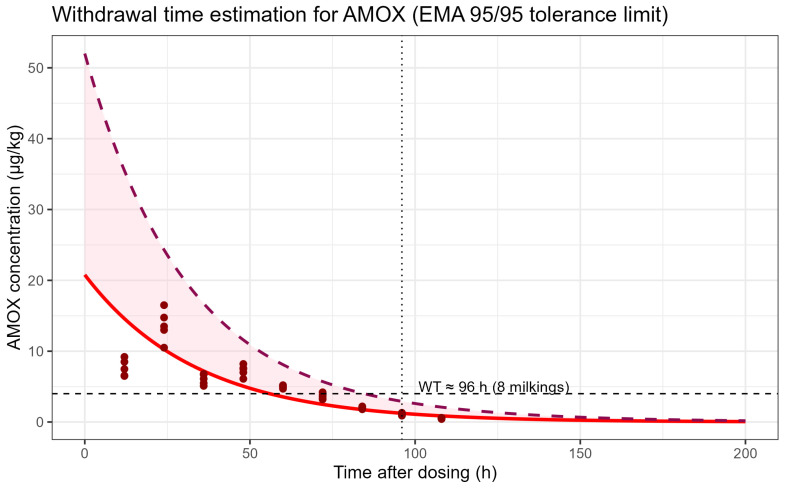
Withdrawal-time estimation for AMOX in buffalo milk based on the EMA 95/95 tolerance limit approach. The solid red line shows the model-fitted mean depletion curve, and the dashed line/shaded band indicates the upper one-sided 95/95 tolerance limit. The horizontal dashed line marks the EU MRL for AMOX in milk (4 µg/kg). The calculated time at which the upper 95/95 tolerance limit first fell below the MRL was 84.7 h; rounded up to the next 12 h milking interval, the reported withdrawal period was 96 h (≈8 milkings). Red dots represent individual observed AMOX concentrations above zero (positive measured values) in milk samples at each sampling time point.

In accordance with EMA guidance, the withdrawal time was defined as the earliest time point at which the upper one-sided 95/95 tolerance limit (upper 95% confidence limit of the 95th percentile of the population) fell below the EU MRL for AMOX in milk (4 µg/kg). Under the conditions of this study, this criterion was first met at 84.7 h after dosing. At this time point, the predicted mean concentration was 1.72 µg/kg and the upper 95/95 tolerance limit was 3.99 µg/kg (Figure 5).

For practical application under a 12 h milking scheme (twice daily), the calculated time was rounded up to the next full milking interval, resulting in a reported withdrawal period of 96 h (≈8 milkings).

Taken together, the depletion data show that AMOX itself is eliminated from buffalo milk relatively rapidly (non-detectable from the tenth milking onward), whereas its major metabolites—particularly 2,5-DKP—persist for longer at low levels (detectable up to the thirteenth milking), consistent with their longer apparent terminal half-lives (AMOX 23.5 h vs. 32.0 h for AMA and 52.8 h for 2,5-DKP; Table 2). This pattern underlines the im-portance of considering both the parent compound and its metabolites when establishing species-specific withdrawal periods and assessing residue-related risks in buffalo milk.

## 4. Discussion

This study characterizes the milk depletion profile of amoxicillin (AMOX) and its two major metabolites, amoxicillinic (amoxicillolic) acid (AMA) and amoxicillin diketopiperazine-2′,5′-dione (2,5-DKP), in Anatolian water buffaloes after intramuscular administration. Overall, AMOX reached its highest milk concentration at the second milking (24 h) and then declined steadily; mean concentrations fell below the European Union MRL (4 µg/kg) by the sixth milking (~72 h). In contrast, both metabolites remained detectable for longer (up to the thirteenth milking vs. AMOX to the ninth; Supplementary Table S4), with 2,5-DKP showing the slowest decline as reflected by its longer half-life (52.8 h vs. 23.5 h for AMOX; Table 2). Taken together, the depletion curves indicate that, in this setting, metabolites can persist in milk after the parent compound has decreased to low or non-quantifiable levels.

From a regulatory standpoint, mean AMOX concentrations declined below the EU MRL (4 µg/kg) [21] by the sixth milking (~72 h), and all individual samples were below the MRL from the seventh milking onward For withdrawal-time estimation, the EMA-recommended 95/95 tolerance limit approach was applied [34]. The EMA-recommended 95/95 tolerance limit approach yielded a calculated withdrawal time of 84.7 h. Because withdrawal periods are implemented on a milking schedule, this value was rounded up to the next 12 h milking interval, resulting in a practical withdrawal period of 96 h (≈8 milkings) to ensure compliance with the MRL with high statistical confidence. Comparisons with cow studies [24,39] should be interpreted cautiously because withdrawal times can vary with formulation, route of administration (intramuscular vs. intramammary), disease status and milking management.

For on-farm decision-making under twice-daily milking, the key operational message is simple: after a single 15 mg/kg intramuscular dose of this long-acting amoxicillin product, milk should be withheld for at least 96 h (about eight milkings). In addition, because AMA and 2,5-DKP persisted longer than AMOX in our dataset, metabolite-inclusive LC–MS/MS confirmation can be informative when interpreting late-time or borderline samples in residue-monitoring workflows.

Beyond dairy cows, milk from other farm species—particularly goats, sheep and camels—contributes to regional diets and specialty dairy products. This broader perspective matters because residue depletion is not always transferable across species. In lactating goats treated therapeutically with intramuscular amoxicillin, Schroeder et al. [40] reported that AMOX was usually detectable for only 1 day and at most 3 days after the end of treatment, and that all samples tested negative for residues before the end of the withdrawal period. These observations are compatible with our buffalo results, but they also highlight that “days-to-clear” should not be assumed to be identical across species.

At the same time, small-ruminant literature emphasizes that depletion and withdrawal recommendations depend strongly on formulation and route (intramuscular vs. intramammary), disease status and milking management, and the sensitivity of the analytical method used to define the “last positive” time point. A recent review by Richards et al. [41] compiled residue depletion data for antibiotics in sheep and goats and underscored the practical challenge, which is that these species often have limited availability of labeled products, increasing reliance on extra-label use and making species-specific residue evidence particularly valuable for veterinarians and producers. Mechanistic work using comparative physiologically based kinetic (PBK) modeling (oxytetracycline as a case study) likewise supports that drug excretion into milk is driven by the compound’s physicochemical properties together with animal physiology and milk composition, so cross-species extrapolation can be uncertain [42]. In some dairy species, residues may persist far longer than expected from bovine experience; for example, in lactating dromedary camels treated intramuscularly with procaine penicillin G/dihydrostreptomycin, penicillin residues were reported to remain above the MRL even 56 days post-treatment, illustrating the risk of adopting bovine withdrawal periods for non-bovine milks [43]. From a practical standpoint, reliable, species-appropriate withdrawal intervals help avoid both residue violations (and associated consumer risk) and unnecessarily discarding milk—exactly the balance that buffalo-specific depletion data aim to support.

Routine residue monitoring tools may also require species-specific validation: in buffalo milk, Büthe et al. showed that the Brilliant Black Reduction Test can achieve detection limits at or below European MRLs, but that incubation times required adjustment compared with cow milk [44].

A further contribution of this work is the analytical method optimized for buffalo milk, a matrix typically richer in fat and protein than cow milk. The achieved sensitivity (LOD/LOQ: 0.29/0.97 µg/kg for AMOX; 0.72/2.41 µg/kg for AMA; and 0.85/2.83 µg/kg for 2,5-DKP) and the generally high recoveries (typically 86–97% with low RSDs) demonstrate that the sample preparation and LC–MS/MS conditions were suitable for reliable quantification well below the regulatory MRL. When compared numerically with previously reported LC–MS/MS methods, the current workflow shows comparable analytical performance while remaining operationally straightforward (e.g., one-step acetonitrile extraction with repeated centrifugation) (Supplementary Table S3).

The depletion behavior of AMA and 2,5-DKP differed clearly from that of AMOX. Non-compartmental analysis yielded longer apparent half-lives for the metabolites—especially 2,5-DKP—than for AMOX (AMOX 23.5 h; AMA 32.0 h; 2,5-DKP 52.8 h; Table 2), and mixed-effects modeling confirmed different depletion rates among analytes (milking × analyte interaction *p* = 4.63 × 10^−5^; Table 3). This pattern—where the parent drug declines to low levels while major transformation products remain measurable—has also been reported for amoxicillin in other matrices in both controlled stability work and residue-elimination studies [25,26,29]. Practically, this means that decisions based only on the parent compound may understate AMOX-related residues at later milkings, even though current regulatory limits are defined for the parent drug. Where feasible, measuring major metabolites alongside AMOX can therefore provide a more complete picture of residue behavior over time.

This study has limitations that should be considered when generalizing the findings. First, AMOX was administered as a commercial long-acting veterinary medicinal product rather than as the pure active substance; formulation excipients/coformulants may influence the absorption phase and early milk concentrations, and therefore the depletion profile and withdrawal estimate should be interpreted as specific to this formulation, dose, and route of administration. The sample size was small (five animals), all buffaloes were clinically healthy and originated from a single herd, and only one dosing regimen (15 mg/kg intramuscular AMOX) was evaluated. Additional work across different herds, lactation stages, milk yields, and clinical conditions (including mastitis), as well as alternative dosing schemes, would help quantify variability and refine withdrawal recommendations. Moreover, this work focused on milk; parallel measurements in plasma and edible tissues could better describe overall disposition. Finally, further research examining the stability and behavior of AMOX and its metabolites during heat treatment and buffalo-milk processing could clarify how residues evolve along the dairy chain.

## 5. Conclusions

This study provides depletion data for amoxicillin and two major metabolites in Anatolian water buffalo milk after a 15 mg/kg intramuscular dose. Under the conditions tested, amoxicillin peaked at 24 h (second milking) and mean AMOX concentrations declined below 4 µg/kg by the sixth milking (~72 h) and became non-detectable from the tenth milking onward. In contrast, AMA and 2,5-DKP remained detectable up to the thirteenth milking, consistent with their longer apparent half-lives (AMOX 23.5 h vs. 32.0 h for AMA and 52.8 h for 2,5-DKP; Table 2). Modeling based on the EMA 95/95 tolerance limit indicated that, under the conditions tested and for the commercial intramuscular formulation used, a milk withdrawal period of 96 h (≈8 milkings) would keep AMOX concentrations below the EU MRL with high confidence. These findings support the use of species-specific depletion data when defining withdrawal guidance for buffalo milk and motivate further studies across broader production conditions and dosing scenarios, with particular attention to metabolite persistence. Overall, the primary contribution of this study is to provide formulation-specific, species-specific evidence to support safe and evidence-based milk withdrawal recommendations in buffalo dairying.

## Figures and Tables

**Table 1:** Intra-day and inter-day precision of the LC–MS/MS method for AMOX, AMA and 2,5-DKP in buffalo milk (*n* = 9).

	Spiked (µg/kg)	Intra-Day (Mean **±** SD, %)	Intra-Day RSD (%)	Inter-Day (Mean **±** SD, %)	Inter-Day RSD (%)
AMOX	10	93.23 ± 5.69	6.10	92.83 ± 5.99	6.45
	20	93.71 ± 6.31	6.74	91.77 ± 5.45	5.93
	50	90.41 ± 3.46	3.82	86.26 ± 3.88	4.50
AMA	10	95.13 ± 5.98	6.29	92.14 ± 5.19	5.63
	20	96.72 ± 3.09	3.19	94.39 ± 4.54	4.81
	50	92.54 ± 4.80	5.18	91.02 ± 3.92	4.30
2,5-DKP	10	96.13 ± 3.40	3.53	93.60 ± 4.87	5.21
	20	96.26 ± 4.82	5.01	94.88 ± 4.69	4.94
	50	94.85 ± 5.48	5.78	92.13 ± 5.29	5.74

**Table 2:** Mean pharmacokinetic parameters of amoxicillin (AMOX), amoxicillinic acid (AMA) and amoxicillin diketopiperazine-2′,5′-dione (2,5-DKP) in buffalo milk after a single intramuscular dose of AMOX (15 mg/kg). Values are expressed as mean ± SD (*n* = 5). Cmax: maximum observed concentration; Tmax: time to reach maximum concentration; AUC_0_–t: area under the concentration–time curve from time zero to the last sampling time; λ: elimination rate constant; t½: apparent terminal half-life.

Analyte	Cmax (Mean **±** SD)	Tmax (h)	AUC_0_–t (Mean **±** SD)	λ (1/h)	t½ (h)
AMOX	13.65 ± 2.22	24	562.32 ± 58.65	0.02945	23.53
AMA	32.64 ± 4.47	24	1504.68 ± 139.68	0.02163	32.04
2,5-DKP	8.09 ± 1.95	12	416.38 ± 44.24	0.01313	52.80

**Table 3:** Type III ANOVA of fixed effects from the linear mixed-effects model of log_10_-transformed milk concentrations, with milking number and analyte as fixed effects and animal as a random intercept. NumDF: numerator degrees of freedom; DenDF: denominator degrees of freedom; F value: F statistic; Pr(>F): *p*-value associated with the F statistic.

Source	Sum Sq	Mean Sq	NumDF	DenDF	F Value	Pr(>F)
Milking	46.0676	46.0676	1	219	52.356	7.70 × 10^−12^
Analyte	4.7106	2.3553	2	219	2.677	0.0710
Milking × Analyte	18.3884	9.1942	2	219	10.449	4.63 × 10^−5^

## Data Availability

The data presented in this study are available on request from the corresponding author.

## Supplementary Materials

The following supporting information can be downloaded at: https://www.mdpi.com/article/10.3390/ani16060963/s1, Figure S1: Individual amoxicillin (AMOX) concentration–time profiles across consecutive milkings (thin red lines) and the corresponding mean concentration profile (thick red line). Data represent measured AMOX concentrations in milk collected from five buffaloes after intramuscular administration. The plot illustrates the high inter-animal variability around peak concentrations (milking 2), followed by a consistent decline in AMOX levels until depletion to undetectable levels by milking number 10–14; Table S1: Age, parity, total lactation duration, and lactation stage (%) of buffaloes at the start of the experiment; Table S2: Lactation ration provided to Anatolian water buffaloes (kg/head/day); Table S3: Performance characteristics of LC–MS/MS-based methods for determination of AMOX and its metabolites in different food matrices; Table S4: Concentrations of AMOX, AMA and 2,5-DKP in buffalo milk across successive milkings (mean ± SD, min–max, coefficient of variation); Table S5: Individual pharmacokinetic parameters (Cmax, Tmax and AUC_0_–t) of AMOX, AMA and 2,5-DKP in buffalo milk (*n* = 5 buffaloes).

## Abbreviations

The following abbreviations are used in this manuscript:

AMOX	Amoxicillin
AMA	Amoxicillinic (amoxicillolic) acid
2,5-DKP	Amoxicillin diketopiperazine-2′,5′-dione
AUC_0_–t	Area under the concentration–time curve from time zero to the last sampling time
Cmax	Maximum observed concentration
CV	Coefficient of variation
ESI	Electrospray ionization
EU	European Union
LC–MS/MS	Liquid chromatography–tandem mass spectrometry
LOD	Limit of detection
LOQ	Limit of quantification
MRL	Maximum residue limit
MRM	Multiple reaction monitoring
PK	Pharmacokinetic(s)
RSD	Relative standard deviation
SD	Standard deviation
t½	Apparent terminal half-life
Tmax	Time to reach maximum concentration
