# Supplementary material for: Depletion of Amoxicillin and Its Major Metabolites in Anatolian Water Buffalo Milk After Intramuscular Administration
Source: Animals (Basel). 2026 Mar 19;16(6):963. doi: 10.3390/ani16060963 (PMC13023250; doi:10.3390/ani16060963)
Supplement: Supplementary file 1 [file animals-16-00963-s001.zip › animals-4104832-supplementary.pdf]

Supplementary Materials

Depletion of amoxicillin and its major metabolites in Anatolian water buffalo milk after intramuscular administration

CONTENTS

Figure S1. Individual amoxicillin (AMOX) concentration–time profiles across consecutive milkings (individual animals and mean profile)……………………………………………………………………1

Table S1. Age, parity, total lactation duration, and lactation stage (%) of buffaloes at the start of the experiment……………………………………………………………………………………………2

Table S2. Lactation ration provided to Anatolian water buffaloes (kg/head/day)………………………2

Table S3. Performance characteristics of LC–MS/MS-based methods for determination of AMOX and its metabolites in different food matrices (LOD, LOQ, recovery/bias; literature comparison)…………3

Table S4. Concentrations of AMOX, AMA and 2,5-DKP in buffalo milk across successive milkings (mean ± SD, min–max, coefficient of variation; n = 5)……………………………………………………4

Table S5. Individual pharmacokinetic parameters (Cmax, Tmax and AUC_0–t) of AMOX, AMA and 2,5-DKP in buffalo milk(n = 5 buffaloes)……………………………………………………………5.

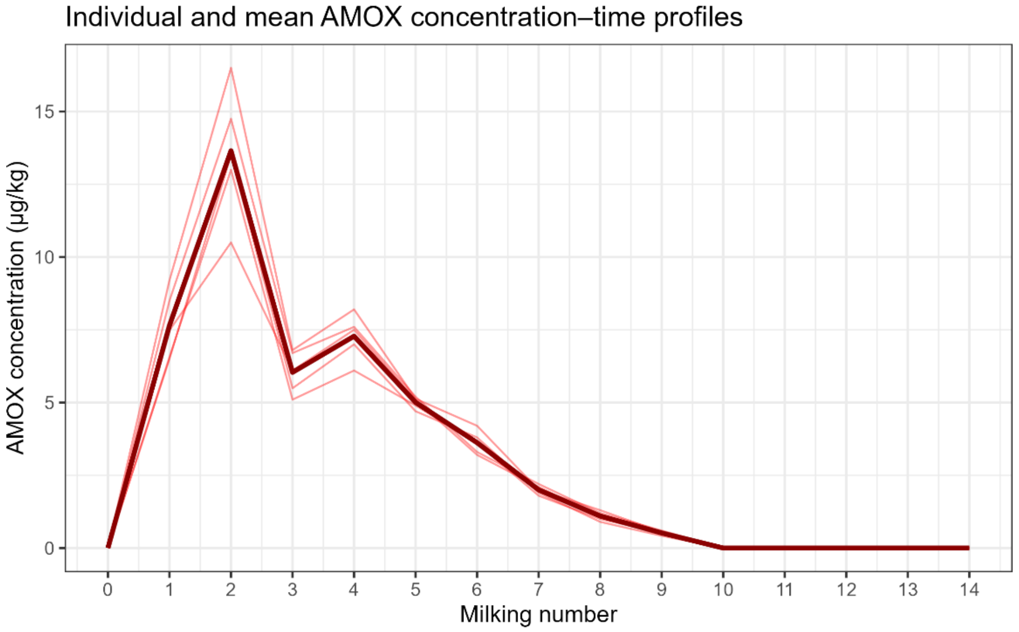

Figure S1. Individual amoxicillin (AMOX) concentration–time profiles across consecutive milkings (thin red lines) and the corresponding mean concentration profile (thick red line). Data represent measured AMOX concentrations in milk collected from five buffaloes after intramuscular administration. The plot illustrates the high inter-animal variability around peak concentrations (milking 2), followed by a consistent decline in AMOX levels until depletion to undetectable levels by milking number 10–14.

Table S1. Age, parity, total lactation duration, and lactation stage (%) of buffaloes at the start of the experiment

Animals	Age (years)	Parity	Total lactation duration (days)	Lactation completed at start (%)
Buffalo 1	5.0	3	255	52.2
Buffalo 2	4.9	3	273	55.3
Buffalo 3	4.8	2	226	78.8
Buffalo 4	3.2	1	337	52.5
Buffalo 5	3.0	1	156	69.2

Table S2. Lactation ration provided to Anatolian water buffaloes (kg/head/day).

Feed Component	Amount (kg/head/day)
Straw	3.0
Alfalfa hay	4.0
Barley	3.7
Dairy concentrate feed	5.0
Sodium bicarbonate	0.15

Table S3. Performance characteristics of LC–MS/MS-based methods for determination of AMOX and its metabolites in different food matrices.

Method	Matrix	Analyte	LOD (μg/kg)	LOQ (μg/kg)	Recovery / bias (%)*	Reference
LC–MS/MS	Buffalo milk	AMOX	0.29	0.97	86.3–93.7	Current study
		AMA	0.72	2.41	91.0–96.7	
		2,5-DKP	0.85	2.83	92.1–96.3	
UHPLC–MS/MS	Cow milk	AMOX	1.0	5.0	−1.9 to +5.0†	Liu et al. [35]
		AMA	1.0	5.0	+4.7 to +7.1†	
		2,5-DKP	0.2	5.0	+0.2 to +2.8†	
LC–MS/MS	Egg	AMOX	0.6	1.8	85.2–88.7	Sun et al. [37]
		AMA	0.5	1.5	80.2–84.2	
		2,5-DKP	0.3	0.9	90.7–91.5	
LC–MS/MS	Chicken muscle	AMOX	0.52	4.10	90.8–106.3	Zhang et al. [38]
		AMA	1.04	4.10	90.5–94.8	
		2,5-DKP	0.15	0.45	95.2–104.5	
	Chicken liver	AMOX	0.85	3.60	92.9–97.2	
		AMA	1.65	6.40	83.1–97.7	
		2,5-DKP	0.30	0.90	93.5–101.0	
	Chicken kidney	AMOX	1.20	4.50	92.4–102.9	
		AMA	2.20	8.50	95.1–103.6	
		2,5-DKP	0.46	1.38	99.9–101.4	
LC–MS/MS	Pig kidney	AMOX	1.7	25	−3.3 to +9.1†	Reyns et al. [36]
		AMA	7.1	25	−2.8 to −0.6†	
		2,5-DKP	2.7	25	−14.3 to −5.7†	
	Pig liver	AMOX	3.5	25	−7.0 to +7.0†	
		AMA	14.2	25	−3.0 to +1.6†	
		2,5-DKP	1.6	25	−2.6 to +4.8†	
	Pig muscle	AMOX	1.5	25	+0.2 to +2.0†	
		AMA	11.1	25	+3.8 to +8.2†	
		2,5-DKP	0.9	25	−6.6 to −0.8†	
	Pig fat	AMOX	1.7	25	+3.0 to +5.1†	
		AMA	11.6	25	−2.6 to +1.5†	
		2,5-DKP	0.9	25	+1.9 to +3.6†	

* Recovery ranges for the current study, Sun et al. [37] and Zhang et al. [38].
† For Liu et al. [35] and Reyns et al. [36], values represent percent bias (trueness) rather than classical recovery; they express deviation from the fortified concentration and are therefore not directly comparable with recovery data reported in other studies.

Table S4. Concentrations of AMOX, AMA and 2,5-DKP in buffalo milk across successive milkings (mean ± SD, min–max, coefficient of variation).

Analyte	Milking	n	Mean ± SD	Min–Max	CV (%)
AMOX	0	5	<LOD	–	–
	1	5	7.648 ± 1.19	6.5–9.2	15.56
	2	5	13.65 ± 2.22	10.5–16.5	16.26
	3	5	6.038 ± 0.74	5.1–6.8	12.29
	4	5	7.28 ± 0.79	6.1–8.2	10.79
	5	5	5.006 ± 0.20	4.7–5.2	4.07
	6	5	3.62 ± 0.40	3.2–4.2	11.12
	7	5	2.00 ± 0.16	1.8–2.2	7.91
	8	5	1.10 ± 0.16	0.9–1.3	14.37
	9	5	0.518 ± 0.066	0.42–0.6	12.83
	10–14	5	<LOD	0–0	–
AMA	0	5	<LOD	0–0	–
	1	5	21.24 ± 2.57	17.56–24.56	12.08
	2	5	32.64 ± 4.47	28.16–39.77	13.70
	3	5	13.66 ± 1.19	11.94–15.18	8.69
	4	5	12.70 ± 1.43	10.95–14.92	11.23
	5	5	10.99 ± 1.12	9.43–12.51	10.17
	6	5	10.08 ± 0.82	8.94–11.24	8.10
	7	5	7.546 ± 0.99	6.09–8.78	13.15
	8	5	5.222 ± 0.40	4.82–5.78	7.69
	9	5	3.988 ± 0.61	3.19–4.67	15.37
	10	5	2.342 ± 0.41	2.05–3.06	17.39
	11	5	1.976 ± 0.05	1.89–2.01	2.49
	12	5	1.748 ± 0.11	1.63–1.88	6.18
	13	5	1.258 ± 0.34	0.73–1.63	26.85
	14	5	<LOD	0–0	–
2,5-DKP	0	5	<LOD	0–0	–
	1	5	8.086 ± 1.95	6.01–10.8	24.09
	2	5	4.576 ± 1.13	3.17–6.27	24.66
	3	5	3.948 ± 0.22	3.58–4.15	5.54
	4	5	3.446 ± 0.08	3.37–3.57	2.22
	5	5	2.816 ± 0.09	2.68–2.90	3.22
	6	5	2.29 ± 0.03	2.26–2.32	1.23
	7	5	1.866 ± 0.11	1.74–1.99	6.00
	8	5	1.56 ± 0.03	1.53–1.61	2.03
	9	5	1.45 ± 0.06	1.37–1.53	4.36
	10	5	1.322 ± 0.02	1.29–1.35	1.64
	11	5	1.23 ± 0.02	1.20–1.26	1.99
	12	5	1.138 ± 0.01	1.13–1.15	0.74
	13	5	0.97 ± 0.02	0.94–0.99	1.93
	14	5	<LOD	0–0	–

Table S5. Individual pharmacokinetic parameters (Cmax, Tmax and AUC_0–t) of AMOX, AMA and 2,5-DKP in buffalo milk (n = 5 buffaloes).

Animal	Analyte	Cmax	Tmax (h)	AUC_0–t
1	AMOX	10.5	24	522.96
	AMA	32.16	24	1660.56
	2,5-DKP	10.8	12	478.68
2	AMOX	13.5	24	530.52
	AMA	39.77	24	1610.88
	2,5-DKP	9.34	12	439.44
3	AMOX	14.75	24	600.6
	AMA	33.36	24	1530.12
	2,5-DKP	7.41	12	406.2
4	AMOX	16.5	24	646.56
	AMA	28.16	24	1376.4
	2,5-DKP	6.87	12	394.32
5	AMOX	13	24	510.96
	AMA	29.75	24	1345.44
	2,5-DKP	6.01	12	363.24
